# Extraction and inhibition of enzymatic activity of botulinum neurotoxins /B1, /B2, /B3, /B4, and /B5 by a panel of monoclonal anti-BoNT/B antibodies

**DOI:** 10.1186/1471-2091-12-58

**Published:** 2011-11-15

**Authors:** Suzanne R Kalb, Wanda I Santana, Isin N Geren, Consuelo Garcia-Rodriguez, Jianlong Lou, Theresa J Smith, James D Marks, Leonard A Smith, James L Pirkle, John R Barr

**Affiliations:** 1 Centers for Disease Control and Prevention, National Center for Environmental Health, Division of Laboratory Sciences, 4770 Buford Hwy, N.E., Atlanta, GA 30341, USA; 2 Department of Anesthesia, University of California at San Francisco, Rm. 3C-38, San Francisco General Hospital, 1001 Potrero Ave., San Francisco, CA 94110, USA; 3 Integrated Toxicology, United States Army Medical Research Institute of Infectious Diseases (USAMRIID), Fort Detrick, MD 21702, USA; 4 Office of the Chief Scientist, Medical Research and Materiel Command (MRMC), Fort Detrick, MD 21702, USA

## Abstract

**Background:**

Botulism is caused by botulinum neurotoxins (BoNTs), extremely toxic proteins which can induce respiratory failure leading to long-term intensive care or death. Treatment for botulism includes administration of antitoxins, which must be administered early in the course of the intoxication; therefore, rapid determination of human exposure to BoNT is an important public health goal. In previous work, our laboratory reported on Endopep-MS, a mass spectrometry-based activity method for detecting and differentiating BoNT/A, /B, /E, and /F in clinical samples. We also demonstrated that antibody-capture is effective for purification and concentration of BoNTs from complex matrices such as clinical samples. However, some antibodies inhibit or neutralize the enzymatic activity of BoNT, so the choice of antibody for toxin extraction is critical.

**Results:**

In this work, we evaluated 24 anti-BoNT/B monoclonal antibodies (mAbs) for their ability to inhibit the *in vitro *activity of BoNT/B1, /B2, /B3, /B4, and /B5 and to extract those toxins. Among the mAbs, there were significant differences in ability to extract BoNT/B subtypes and inhibitory effect on BoNT catalytic activity. Some of the mAbs tested enhanced the *in vitro *light chain activity of BoNT/B, suggesting that BoNT/B may undergo conformational change upon binding some mAbs.

**Conclusions:**

In addition to determining *in vitro *inhibition abilities of a panel of mAbs against BoNT/B1-/B5, this work has determined B12.2 and 2B18.2 to be the best mAbs for sample preparation before Endopep-MS. These mAb characterizations also have the potential to assist with mechanistic studies of BoNT/B protection and treatment, which is important for studying alternative therapeutics for botulism.

## Background

Botulism is a disease which can be fatal if untreated and is caused by exposure to any one of the highly toxic protein family known as botulinum neurotoxins (BoNTs). *In vivo*, BoNT cleaves proteins necessary for nerve signal transmission. This enzymatic cleavage results in the inhibition of the nerve impulse, leading to flaccid paralysis of the victim which can affect the lungs and may necessitate ventilator support. Treatment of the botulism patient involves administration of therapeutic immunoglobulin and is most effective when administered within 24 h of toxin exposure [1]. Due to the extreme toxicity, global availability, and ease of preparation of BoNT, it is considered a likely agent for bioterrorism [2].

Previously, our laboratory reported in several publications on the development of the Endopep-MS method as an assay for BoNT detection and serotype differentiation [3,4]. This method can detect all seven known BoNT serotypes and involves incubating BoNT with a peptide substrate that mimics each toxin's natural *in vivo *neuronal protein target. The presence of a particular BoNT serotype is demonstrated by mass spectrometric detection of the peptide cleavage products corresponding to their specific toxin-dependent location. Endopep-MS currently uses an antibody-affinity concentration/purification step before the enzymatic reaction with the substrate, and the choice of antibody is critical for the success of this assay [5]. We previously reported that polyclonal anti-BoNT binding could interfere with the activity of BoNT as measured by Endopep-MS [5]. We also reported on the success of using monoclonal (mAb) anti-BoNT/A to detect multiple subtypes of BoNT/A [6,7].

Similar to the other BoNT serotypes, BoNT/B consists of a heavy chain (HC) of approximately 100,000 daltons and a light chain (LC) of about 50,000 daltons. The heavy chain is mainly responsible for both receptor binding by its C-terminal (CT) binding domain [8,9] (H_C_) and the delivery of the catalytic light chain (LC) to its target inside the neuron by way of its N-terminal translocation domain (H_N_)[10]. Although the LC is responsible for the specific toxicity, it requires the heavy chain to enter the target cell and produce this toxic activity *in vivo*. As with most of the other BoNT serotypes, BoNT/B exhibits genetic and amino acid variance within the serotype, and this variance is defined as a subtype. BoNT/B is currently defined as consisting of the /B1, /B2, /B3, /B4, /B5, and /B6 subtypes. [11,12]. At the amino acid composition level, the variance among all the BoNT/B is 7% or less, but this degree of variance can affect binding of the toxin to some of the anti-BoNT/B mAbs as shown before [13]. So, it is important to select cross reactive mAbs which are able to detect all toxin subtypes, because an outbreak of BoNT/B botulism may be attributed to more than just the familiar "common" subtype.

Previously, our laboratory demonstrated that the Endopep-MS assay can be used to detect all currently known available subtypes of BoNT/B [7,14]. The goal of this work is to evaluate a panel of mAbs for their inhibitory and extraction abilities, thereby optimizing assay sensitivity with all BoNT/B subtypes available to us for testing. Here, we evaluated a panel of 24 fully human monoclonal anti-BoNT/B mAbs for their ability to inhibit the *in vitro *light chain activity of BoNT/B1, /B2, /B3, /B4, or /B5. BoNT/B6 was unavailable to us for testing. Additionally, we evaluated the same antibody panel for their ability to extract any of the available subtypes of BoNT/B. Our data show that there were significant differences among those mAbs in their ability to extract different BoNT/B subtypes, and their inhibitory effects on BoNT/B catalytic activity. Surprisingly, some of the mAbs appeared to enhance the light chain enzymatic activity of some subtypes of BoNT/B, a phenomenon that has been reported for BoNT/A, but not yet for BoNT/B [15]. Such differences could be explained in part through analyzing the epitopes of the mAbs and the amino acid sequences of each subtype of BoNT/B. Our results indicate which mAbs have the optimal properties for use in the Endopep-MS detection of BoNT/B.

## Methods

### Materials

Botulinum neurotoxin is very toxic and must be handled with extreme care and appropriate safety measures. All neurotoxins were handled in a level 2 biosafety cabinet equipped with HEPA filters. Commercially purified BoNT/B1 (strain Okra) was purchased from Metabiologics (Madison, WI). Crude culture supernatants representing various BoNT/B subtypes [11] were produced by incubating subcultures of each strain for 5 days at 30-35°C. Information on the strains used in these studies is listed in Table 1. After centrifugation, supernatants were removed and filtered through 0.22 μm filters. The filtered supernatants were tested for upper limits of toxicity, which indicated that the toxins were all present at concentrations of ≤ 10 μg/mL. Some of the preparations were titered to determine lethality in mouseLD_50 _(mLD_50_)/mL as in reference 6.

**Table 1:** Strain information on culture supernatants used for this study.

Sample	Strain	NCBI accession #
B1 (proteolytic)	Okra	AB232927

B2 (proteolytic)	213B	ABM73972

B3 (proteolytic)	CDC 795	EF028400

B4 (nonproteolytic)	Eklund 17B	EF051570

B5 (bivalent)	An436	EF028397

Dynabeads^® ^Protein G was purchased from Invitrogen (Carlsbad, CA) at 1.3 g/cm^3 ^in phosphate-buffered saline, pH 7.4, containing 0.1% Tween^®^-20 and 0.02% sodium azide. Except where indicated, all chemicals were from Sigma-Aldrich (St. Louis, MO). Peptides were synthesized by Los Alamos National Laboratory (Los Alamos, NM) and are identical to those reported previously [3-5,7,14]. Specifically, the peptide substrate has the sequence LSELDDRADALQAGASQFESSAAKLKRKYWWKNLK and the internal standard peptide (ISTD) has the sequence LSELDDR**A**DALQAGASQ where **A **indicates a +7 mass increase to a naturally occurring alanine.

### Preparation of Monoclonal Antibodies

All the mAbs used in this report were first selected or engineered as antibody leads from scFv or Fab display immune libraries and converted into full-length human IgG1 as previously reported [13,16]. Specifically, mAb A12 and 6A12 were first selected from a scFv phagemid library [17,18] before systematic characterization in the yeast display format. The leads of all other 22 mAbs were generated directly from yeast display immune libraries either in the scFv or Fab format [[13,16], Geren IN, Garcia-Rodriguez C, Lou J, Conrad F, Fan F, et al: Human monoclonal antibodies to botulinum neurotoxin types A and B from immune yeast displayed antibody libraries, submitted]. Some mAbs [19] (B12.1, B12.2, 1B12.3, 1B12.4, B8.1, B6.1, 2B18.1, 2B18.2, 2B18.3, B11E8, 1B22.4, 1B10.1, and 2B25.1) were engineered toward higher affinity or better cross reactivity [20] for most available BoNT/B subtypes, through chain shuffling using a scFv or Fab yeast display system [13,19], before they were converted into the IgG format which consists of the human gamma 1 constant region and the human kappa or lambda constant region. Stable CHO DG44 cell lines were established for each of the antibodies and IgG was purified from cell culture supernatant using a protein G affinity column. The monovalent K_D _was determined for each IgG using kinetic exclusion analysis (Kinexa). All the purified IgG were stored at -70°C until application.

### BoNT/B Inhibition Experiments

A 2-μL solution containing 30 ng of each titered IgG was added to a 2-μL solution containing 25 mLD_50 _of BoNT/B1, or estimated levels of 10-25 mLD_50 _of/B2,/B3,/B4, or/B5. The mixtures were incubated for 1 h at room temperature with no agitation. Then, 16 μL of a reaction mixture (0.05 M Hepes [pH 7.3], 25 mM dithiothreitol, 20 μM ZnCl_2_, 1 mg/mL bovine serum albumin, and 50 pmol/μL of peptide substrate) was added to the mixture. All samples then were incubated at 37°C for 4 h with no agitation. All assays were performed in duplicate and results were averaged.

### BoNT/B Extraction Experiments

The IgG was immobilized and cross-linked to the Dynabeads^® ^Protein G as described in previous publications [5-7,14]. An aliquot of 20 μL of antibody-coated beads was mixed for 1 h with a 0.5-mL solution containing 25 mLD_50 _of BoNT/B1, or estimated levels 10-25 mLD_50 _of/B2-/B5. The solution was prepared by spiking approximately 625 mLD_50 _of either BoNT/B1, /B2, /B3, /B4, or /B5 into 12.5 mL of phosphate-buffered saline with 0.01% Tween (PBST) buffer. After mixing for 1 h with constant agitation at room temperature, the beads were washed twice in 1 mL each of PBST and then washed once in 100 μL of water. The beads were reconstituted in a 20-μL solution containing 0.05 M Hepes (pH 7.3), 25 mM dithiothreitol, 20 μM ZnCl_2_, 1 mg/mL bovine serum albumin, and 50 pmol/μL of peptide substrate. All samples then were incubated at 37°C for 4 h with no agitation. All assays were performed in duplicate and results were averaged.

### Estimation of BoNT/B2-/B5 Activity

As precise mouse LD_50 _titer data were unavailable for the crude toxin extract of BoNT/B2, /B3, /B4, and /B5, we first estimated the activity of these subtypes by testing 25 mLD_50 _of BoNT/B1 in a 2 μL volume in parallel with 2 μL of varying dilutions in water of the crude extract containing BoNT/B2, /B3, /B4, and /B5. All samples were then incubated with 18 μL of a reaction mixture (0.05 M Hepes [pH 7.3], 25 mM dithiothreitol, 20 μM ZnCl_2_, 1 mg/mL bovine serum albumin, and 50 pmol/μL of peptide substrate) for 4 hr at 37C.

### MS Detection

A master mix was created consisting of 9 parts matrix solution (alpha-cyano-4-hydroxy cinnamic acid) at 5 mg/mL in 50% acetonitrile, 0.1% trifluoroacetic acid (TFA), and 10 mM ammonium phosphate) and 1 part ISTD in water at 5 μM. To 18 μL of this master mix, 2 μL of each reaction supernatant were added. We pipetted 0.5 μL of this mixture onto each spot of a 384-spot matrix-assisted laser desorption/ionization (MALDI) plate (Applied Biosystems, Framingham, MA). Mass spectra of each spot were obtained by scanning from 1100 to 5500 *m/z *in MS-positive ion reflector mode on an Applied Biosystems 4800 Proteomics Analyzer (Framingham, MA). The instrument uses a Nd-YAG laser at 355 nm, and each spectrum is an average of 2400 laser shots.

## Results

### Effect of Antibody Addition on BoNT/B Enzymatic Activity

**Figure 1:**
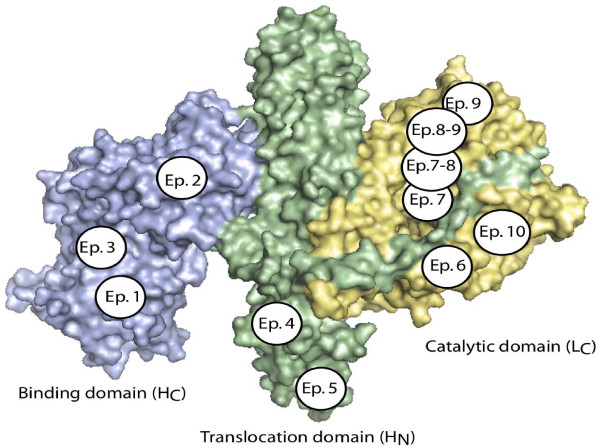
**The X-ray crystal structure of BoNT/B1 with the three functional domains indicated**. Epitopes bound by the 24 mAbs used in this work are indicated. Epitope placement on each domain is arbitrary, except for epitope 5 on the H_N _and epitope 7 on the LC. Epitope 5 is bound by the 1B18 family of mAbs which bind the tip of the H_N_, as indicated (Geren IN, Garcia-Rodriguez C, Lou J, Conrad F, Fan F, et al: Human monoclonal antibodies to botulinum neurotoxin types A and B from immune yeast displayed antibody libraries, submitted). Epitope 7 is bound by 1B10.1, which has been mapped to the indicated location on the LC (data unpublished).

The mAbs evaluated in this work bound 11 different epitopes on the BoNT/B H_C_, H_N_, or LC (Table 2, Figure 1). Because BoNTs consist of two chains, with one chain responsible for enzymatic activity (LC) and another chain (HC) responsible for directing the enzymatically active light chain to its target inside the neuron, antibodies reacting with the LC of the toxin may inhibit the toxin's activity. Therefore, an inhibition experiment was performed in which an equal amount of each antibody was added to the BoNT/B Endopep-MS reaction. The molar concentration of antibody in all cases exceeds the molar concentration of toxin by at least 40- to 70- fold, ensuring that essentially all of the LC is bound by antibody and that antibody activity is evaluated separately from antibody affinity.

**Table 2:** Affinities, domain specificity, and epitopes of 24 human mAbs to BoNT/B.

mAb	BoNT/B epitope	K_D _BoNT/B1 (pM)	K_D _BoNT/B2 (pM)	K_D _BoNT/B3 (pM)	K_D _BoNT/B4 (pM)
6A12	H_C _epitope 1	5150	NB	NB	NB

B12.1	H_C _epitope 1	40.21	15.42	52.28	624.50

B12.2	H_C _epitope 1	346	90.11	NM	NM

1B12.3	H_C _epitope 1	75.73	57.05	68.1	356.73

1B12.4	H_C _epitope 1	15.60	27.11	9.39	399

2B30	H_C _epitope 1	370(scFv)	190(scFv)	NB	NB

B1.1	H_C _epitope 2	477(scFv)	NB	NB	NB

B8.1	H_C _epitope 3	46.03	90.12	NB	NM

A12	H_C _epitope 3	2100	NB	NB	NB

B11E8	H_N _epitope 4	6.59	18.1	15.60	NB

1B18.1	H_N _epitope 5	545	818	976	21

2B18.1	H_N _epitope 5	63	181	91	312

2B18.2	H_N _epitope 5	30.82	83.75	228(scFv)	526(scFv)

2B18.3	H_N _epitope 5	17.4	20.33	128	3108

B6.1	L_C _epitope 6	6.82	9.18	28.5	9.41

2B24	L_C _epitope 6	7.8	10.38	5.44	5.96

1B10.1	L_C _epitope 7	0.33	0.35	1206	0.41

2B27	L_C _epitope 7-8	165.4	560(scFv)	2320(scFv)	77(scFv)

1B22	L_C _epitope 8-9	336.1	319.3	221.1	128.6

1B22.4	L_C _epitope 8-9	139(scFv)	110(scFv)	141(scFv)	129(scFv)

2B25.1	L_C _epitope 8-9	16.69	53.32	9.07	29.53

2B29	L_C _epitope 9	856.7	1020	1610	1290

2B23	L_C _epitope 10	38.07	45.63	54.89	48.23

4B19.1	L_C _epitope 10	176.1	138.5	115.5	194

Affinities were measured for IgG using flow fluorimetry in a KinExA or for yeast displayed scFv using FACS (indicated as scFv after the KD data). Epitope numbers identify mAbs whose binding sites on BoNT/B overlap; mAb with the same epitope number cannot bind BoNT/B simultaneously. "NB" means no binding detected at the highest concentration of toxin (up to 1 μM) tested. "NM" means no measurement was performed for that specific subtype of toxin.

**Figure 2:**
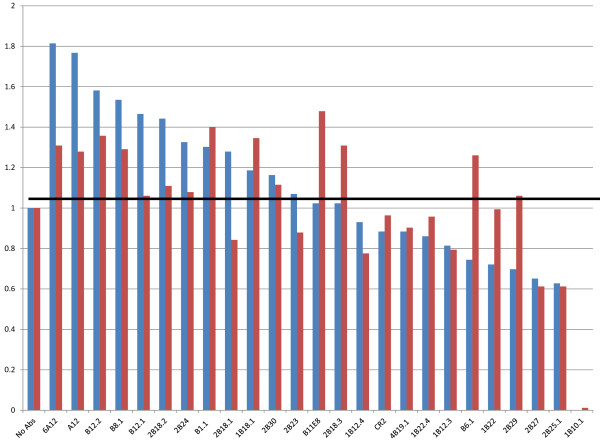
**A graph indicating the % of inhibition in activity of BoNT/B1 (blue) and /B2 (red) following incubation with the antibody panel**. The sample with no antibodies had no inhibition of activity, so the % of inhibition in activity is calculated by dividing the peak area ratio of the peptide cleavage product over the internal standard peptide of the individual antibody by the peak area ratio of the sample with no antibodies.

The inhibition experiment was performed with a panel of 24 mAbs (Table 2) against BoNT/B1, /B2, /B3, /B4, or /B5 toxin complexes. As controls, we also performed the inhibition experiments in the absence of antibodies and in the presence of CR2, an anti-BoNT/A mAb that does not cross-react with toxins in the BoNT B serotype [20]. Table 3 lists the peak area ratios of the native cleavage product over the ISTD obtained from the reaction of these five BoNT/B toxins with all the 25 antibodies and the no-antibody control reaction. An increase in peak area ratio indicates a more enzymatically active toxin because the same level of toxin was used with each mAb. Figure 2 is a graph of the change in enzymatic activity of proteolytic BoNT/B1 and /B2 by antibody binding as calculated through the peak area ratios of each mAb sample compared to the peak area ratio in the absence of any antibodies.

**Table 3:** Peak area ratios of the peptide cleavage product divided by the internal standard peptide obtained from the Endopep-MS reaction of BoNT/B with its peptide substrate in the presence of the antibody panel.

Antibody	BoNT/B1	BoNT/B2	BoNT/B3	BoNT/B4	BoNT/B5
None	0.43	1.65	0.72	0.48	0.73

CR2	0.38	1.59	0.79	0.51	1.08

6A12	0.78	2.16	0.93	0.35	0.87

A12	0.76	2.11	0.83	0.53	0.96

B12.2	0.68	2.24	0.85	0.53	0.89

B8.1	0.66	2.13	0.95	0.48	1.06

B12.1	0.63	1.75	1.03	0.48	1.30

2B18.2	0.62	1.83	0.8	0.56	1.24

2B24	0.57	1.78	0.74	0.53	1.07

B1.1	0.56	2.31	0.81	0.54	1.72

2B18.1	0.55	1.39	0.88	0.61	1.09

1B18.1	0.51	2.22	1.07	0.68	0.45

2B30	0.50	1.84	1.29	0.93	0.58

2B23	0.46	1.45	0.85	0.56	1.32

B11E8	0.44	2.44	0.89	0.51	0.82

2B18.3	0.44	2.16	0.75	0.64	0.76

1B12.4	0.40	1.28	0.89	0.77	1.18

4B19.1	0.38	1.49	0.92	0.71	0.60

1B22.4	0.37	1.58	0.92	0.28	0.80

1B12.3	0.35	1.31	0.68	0.70	1.07

B6.1	0.32	2.08	0.87	0.32	1.49

1B22	0.31	1.64	1.04	0.27	0.71

2B29	0.30	1.75	0.98	0.42	0.65

2B27	0.28	1.01	0.62	0.24	0.72

2B25.1	0.27	1.01	0.87	0.25	0.70

1B10.1	0	0.02	0.19	0.10	0.65

Five different subtypes of BoNT/B were used, and they include /B1, /B2, /B3, /B4, and /B5.

**Figure 3:**
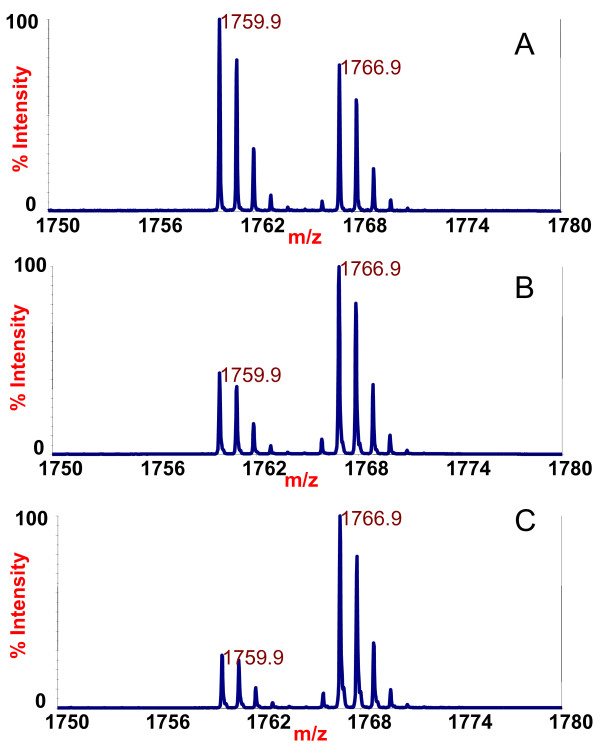
**Mass spectra of the Endopep-MS BoNT/B1 reaction with mAb 6A12 (3A), no antibody (3B), or mAb 2B27 (3C)**. The peptide cleavage product indicating that BoNT/B1 is present to some extent in all cases is at *m/z *1759.9 and the internal standard is present at *m/z *1766.9.

The inhibition studies show that the catalytic activity of BoNT/B1 toxin is inhibited to some degree in the presence of only a portion of the tested mAbs (Table 3, Figure 2). This is in marked contrast to what was observed when studying a panel of BoNT/A mAbs in which the light chain catalytic activity of all subtypes of BoNT/A tested were inhibited in the presence of any mAb tested [6]. Many of the mAbs tested actually increased the LC catalytic activity of BoNT/B1 over the control with no antibodies added to the reaction. For example, after mass spectrometric analysis, it is clear that there was a NT cleavage product at 1759.9 *m/z *in the reaction containing either the mAb 6A12 or no antibody (Figure 3A vs 3B). Both reactions contained the same amount of ISTD at *m/z *1766.9, so by comparison of the size of the 1766.9 peaks with the size of the 1759.9 peaks, we could determine that the amount of NT cleavage product at *m/z *1759.9 was much larger with the mAb 6A12 (Figure 3A) reaction than that with no antibody (Figure 3B). Of note, mAbs that increased LC catalytic activity tended to bind either the H_C _or the H_N _(Table 2 and Figure 2).

These data indicate that mAb 6A12 serves as an activator of the LC enzymatic activity of BoNT/B1 toxin; many of the other mAbs tested also appeared to activate the LC of the toxin to some degree. However, the amount of NT cleavage product is much smaller with the mAb 2B27 reaction (Figure 3C) than those with no antibody (Figure 3B). This indicated that this antibody inhibits the activity of BoNT/B1. The sum of the results for the BoNT/B1 toxin inhibition test indicated that a number of mAbs inhibited the LC catalytic activity, with the most inhibitory antibody being mAb 1B10.1. Most of these inhibitory mAbs bound to the LC. For mAb 1B10.1, there was no cleavage product present at all (Figure 2). In fact, a separate experiment (data not shown) indicated that 30 ng of this antibody appears to inhibit the LC enzymatic activity up to 100 mLD_50 _of BoNT/B1.

The BoNT/B2 protein differs from BoNT/B1 by 4% at the amino acid level. Because it is important to understand whether this level of genetic differences results in inhibition differences with various mAbs, all 24 mAbs were also examined for their enzymatic inhibition of BoNT/B2. The data in Table 3 and Figure 2 show that, despite slight genetic differences, most of the results for inhibition of BoNT/B2 were similar to that for BoNT/B1, with the exception of B6.1, 2B29, and 2B18.1. B6.1 is an inhibitor of BoNT/B1 activity, but appears to be an activator of BoNT/B2 LC activity. mAb 2B29 acts as an inhibitor of BoNT/B1 enzymatic activity; however, it does not have this effect on BoNT/B2 activity.

**Figure 4:**
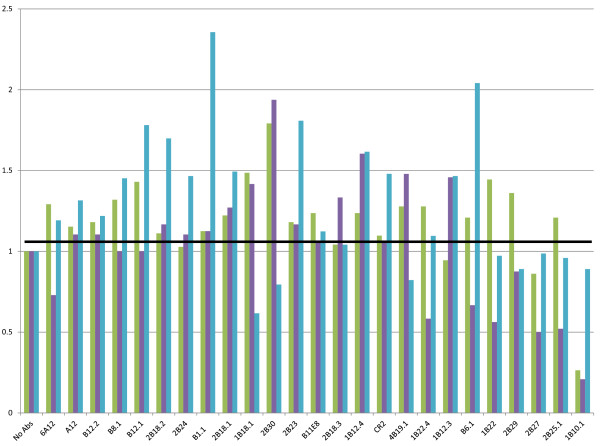
**A graph indicating the % of inhibition in activity of BoNT/B3 (green), /B4 (purple), and /B5 (blue)following incubation with the mAb panel**. The sample with no antibody had no inhibition of activity, so the % of inhibition in activity is calculated by dividing the peak area ratio of the peptide cleavage product over the internal standard peptide of the individual antibody by the peak area ratio of the sample with no antibody.

The proteolytic subtype BoNT/B3 also differs from BoNT/B1 by 4% at the amino acid level, and it differs from BoNT/B2 by 2%, so we examined the effect of all 24 mAbs against BoNT/B3 as well. The results, depicted in Figure 4 and Table 3 are comparable to that observed for BoNT/B1 toxin, with the same exceptions noted on the BoNT/B2 test (B6.1 and 2B29), as described above. Additional notable differences include 1B22 and 1B22.4, which are both inhibitors of BoNT/B1 activity, but had no effect on BoNT/B3 activity.

Non-proteolytic BoNT/B4 is the most dissimilar among all the BoNT/B subtypes and differs from BoNT/B1 by 7% at the amino acid level. This toxin, like all other BoNT/B, is produced as a single chain neurotoxin, but unlike other BoNT/B, it remains as a single chain as opposed to being cleaved into a dichain toxin. Incubating this toxin with the panel of 24 mAbs yielded much the same results as with the other BoNT/B subtypes. This is seen in Figure 4 and Table 3. As with BoNT/B2 and/B3, mAbs B6.1 and 2B29 did not appear to inhibit this toxin subtype. Additionally, mAbs 2B25.1 and 2B27 also did not appear to inhibit the activity of BoNT/B4 whereas these mAbs inhibited the activity of other BoNT/B subtypes.

BoNT/B5, produced by bivalent *Clostridium *strains, differs from BoNT/B1 by 4% at the amino acid level. The only differences in activity of this toxin subtype against the panel of 24 mAbs compared to the results from the BoNT/B1 activity test were noticed with mAbs B6.1 and 1B10.1, as seen in Figure 4 and Table 3. B6.1 had no inhibition effect on the activity of BoNT/B5. Interestingly, mAb 1B10.1 also had no inhibition effect on the activity of BoNT/B5, perhaps due to its lower affinity for BoNT/B5 compared to the other BoNT/B subtypes (unpublished data).

### BoNT/B extraction efficiency

**Figure 5:**
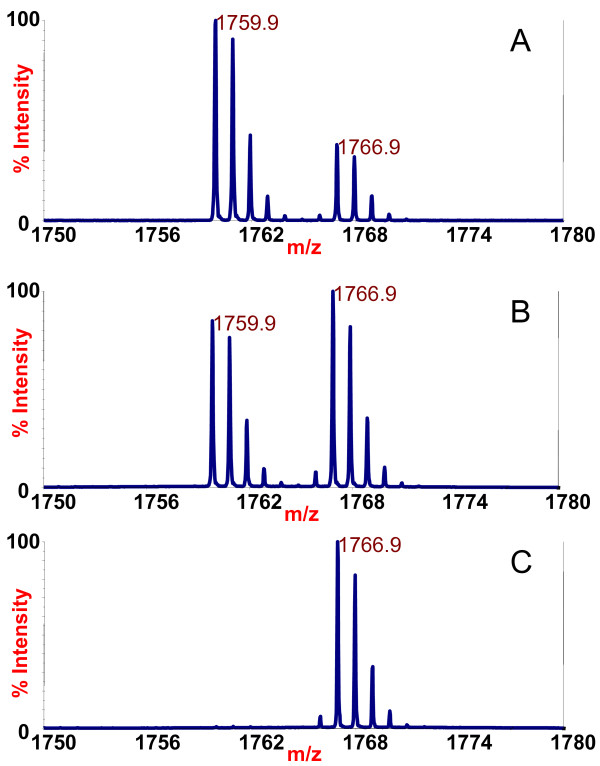
**Mass spectra of the Endopep-MS botulinum neurotoxin B1 reaction after extraction of the toxin with mAb B12.2 (5A), no antibody (5B), or mAb B1.1 (5C)**. The peptide cleavage product indicates the presence of BoNT/B in 5A and 5B, but not 5C, at *m/z *1759.9 and the internal standard is present in all spectra at *m/z *1766.9.

After determining which antibodies were inhibitory toward the catalytic activity of BoNT/B1-/B5, we wanted to examine the ability of any of the panel of 24 mAbs in our assay to extract BoNT/B1-/B5 in the Endopep-MS assay. Extraction assessed both the ability of the mAb to bind BoNT/B, reflected by the mAb affinity, as well as the inhibition of BoNT/B by the mAbs. All mAbs were used to extract the same level of BoNT/B from a buffer solution. After extraction, the toxins on the beads were added to identical reaction mixtures containing peptide substrate. Upon mass spectrometric analysis, it was apparent that the mAb B12.2 (Figure 5A), non-extracted (Figure 5B), and mAb B1.1 (Figure 5C) extracted samples contained the internal standard at *m/z *1766.9, but only the B12.2 mAb-extracted sample and the non-extracted control contained N-terminal cleavage product at *m/z *1759.9. Comparing the NT products with ISTDs shows that the mAb B12.2 antibody-extracted sample contains more N-terminal cleavage product than the mAb B1.1 antibody-extracted sample or the non-extracted control. Because all samples contained the same amount of internal standard, the generation of a higher level of cleavage product indicated a greater level of toxin, a higher activity of toxin, or possibly both for mAb B12.2 compared to mAb B1.1.

Table 4 contains the peak area ratios of the cleavage product over the ISTD for 24 BoNT/B mAbs used to extract BoNT/B1, /B2, /B3, /B4, or /B5. mAbs B1.1 and 1B10.1 yielded no cleavage product, and mAb 1B22.4 yielded minimal cleavage product. As with the inhibition studies, we also wanted to check extraction efficiency differences introduced by genetic differences among the five BoNT/B subtypes. A number of mAbs that had good extraction efficiency for BoNT/B1 also had good extraction efficiency for BoNT/B2-/B5. These include mAbs 1B18.1, 2B18.1, 2B18.2, 2B18.3, B12.2, and 2B23. Some antibodies, like mAbs B1.1, 1B10.1, or 1B22.4, had either poor extraction efficiency or inhibited the enzymatic activity of all five BoNT/B subtypes tested. Interestingly, some antibodies (such as mAb B11E8 and 2B30) had good extraction efficiency for four of the five BoNT/B subtypes tested and appeared to activate these toxins, but had poor extraction efficiency for BoNT/B4 which is produced by a nonproteolytic bacteria strain. In the case of B11E8, this can be explained by its inability to bind to the BoNT/B4 subtype (Table 2).

**Table 4:** Peak area ratios of the peptide cleavage product divided by the internal standard peptide obtained from the Endopep-MS reaction of BoNT B after its extraction by the antibody panel.

Antibody	BoNT/B1	BoNT/B2	BoNT/B3	BoNT/B4	BoNT/B5
None	1.02	1.62	0.46	0.14	1.26

B11E8	4.03	1.68	5.71	0.002	1.84

1B12.3	3.58	1.68	4.31	0.09	1.85

2B18.2	3.47	1.25	5.58	0.29	1.60

B8.1	3.30	1.44	0.54	0.14	0.009

B12.2	3.30	1.70	4.14	0.15	1.35

2B30	3.21	1.27	4.44	0.002	1.35

B12.1	3.18	1.89	5.51	0.19	1.65

1B18.1	3.14	1.23	4.98	0.19	1.51

2B18.3	3.14	1.17	5.14	0.17	1.39

2B18.1	3.04	1.15	3.94	0.22	1.62

1B12.4	2.92	1.11	3.35	0.13	2.26

2B24	2.76	1.01	2.93	0.19	1.60

2B23	2.68	1.18	3.24	0.11	1.19

2B25.1	2.38	0.72	1.69	0.05	0.69

A12	2.36	1.47	0.99	0.12	0

B6.1	2.35	1.12	3.16	0.15	1.51

4B19.1	2.31	1.25	3.93	0.21	2.11

6A12	1.39	1.09	2.50	0.07	1.18

1B22	0.95	0.72	3.79	0.03	0.95

2B27	0.92	0.56	1.29	0.05	1.12

2B29	0.88	0.80	1.90	0.03	0.87

1B22.4	0.54	0.21	0.32	0.009	0.06

B1.1	0	0	0	0	0.002

1B10.1	0	0	0	0	0.11

Five different subtypes of BoNT/B were used, and they include /B1, /B2, /B3, /B4, and /B5.

## Discussion

After examining a panel of BoNT/A [6] and BoNT/B mAbs (this study) we found that there are significant differences in the ability of the mAbs to inhibit or activate BoNT. Most importantly, we did not observe activation of the LC activity by any of the anti-BoNT/A mAbs, whereas we did observe this with a number of BoNT/B mAbs. Interestingly, in this study, the majority of the mAbs that enhanced the enzymatic activity of the BoNT/B subtypes bound the HC portion of the toxin, which is not enzymatically active.

The phenomenon of toxin activation by antibody binding is not a novel one [15], and it is thought that some mAbs induce a conformational change upon the structure of the toxin after binding so that the toxin is induced into an optimal conformation to bind the substrate. It is highly possible that this induced-conformational change was responsible for the increase in BoNT/B LC enzymatic activity with some mAbs, particularly those which did not bind near the enzymatic active site. It is also important that although a mAb could be an activator of LC activity in an enzymatic assay, it does not mean that this mAb would enhance *in vivo *toxicity.

Not all of the anti-BoNT/B mAbs were LC activators. For example, mAb 1B10.1 inhibited the LC activity of four of the five BoNT/B subtypes. Antibody 1B10.1 bound the LC of the toxin (Table 2, Figure 1), and residues R121, R122, N177, H179, F180, R183, E184, D244, and D245 of BoNT/B contribute to the binding of 1B10.1 as mapped [21] using yeast displayed BoNT/B LC (C. Garcia and J.D. Marks, unpublished data). The active site of BoNT/B was centered around residue 231, which is quite close to the epitope for the binding of mAb 1B10.1. It is highly probable that upon binding of 1B10.1, BoNT/B toxin is no longer able to access the peptide substrate, and is therefore inactive upon the peptide substrate. This explains the ability of 1B10.1 to inhibit the enzymatic activity of most BoNT/B subtypes. Looking at the amino acid similarities of the BoNT/B subtypes in these residues, we found that only one mutation existed--the aspartic acid in position 244, which is negatively charged, is mutated to neutral asparagine in BoNT/B5. This mutation may explain the lower affinity of mAb 1B10.1 for BoNT/B5 and its lack of inhibition.

Another inhibitory antibody is 2B27, which bound the LC of the toxin. The 2B27 epitope overlaps the epitope of mAb 1B10.1 (Table 2 and Figure 1). This proximity to the active site of the toxin explains its ability to inhibit the LC activity of this toxin. In fact, most of the other inhibitory antibodies are also LC binders, presumably because these mAbs interfere with the binding of the substrate to the toxin. It is important, however, that not all LC binders inhibit BoNT/B1 enzymatic activity. mAb 2B23 bound the LC, but had virtually the same response in enzymatic activity as the control with no antibody. Although mAb 2B23 bound the LC (Table 2, Figure 1) the epitope seems to be farther away from the enzymatic active site, so it does not have an inhibitory effect on the toxin's activity.

B6.1 was inhibitory for some BoNT/B subtypes, but acted as an activator of LC activity for other subtypes. mAb B6.1 bound the LC of toxin on epitope 6; however, it did not bind in close proximity to the enzymatic active site (Figure 1). Therefore, it is possible that B6.1 binding inhibits some BoNT/B not by B6.1 itself blocking the peptide substrate from the active site, but rather by the induced conformational change upon that toxin. This would result in an altered quaternary structure of BoNT/B1 and /B4 which has difficulty contacting the peptide substrate, while the changed conformation of BoNT/B2, /B3, and /B5 upon mAb B6.1 binding might have easier access to the substrate, thereby serving as an activator of LC activity for BoNT/B2, /B3, and /B5. This phenomenon could also explain the activity of 2B29 and 2B18.1, which also serve as inhibitors for some subtypes of BoNT/B and LC activators for other subtypes.

The extraction efficiency experiments also shed some light on the interaction of these mAbs with the different BoNT/B subtypes. Three mAbs which had poor extraction efficiency were 1B10.1, 1B22.4, and B1.1. Because the inhibitory experiments showed that the mAbs 1B10.1 and 1B22.4 inhibited the enzymatic activity of BoNT/B, the decreased cleavage products after extraction with these mAbs were likely due to inhibition of activity rather than poor extraction efficiency. In contrast, the mAb B1.1 was a moderate to strong LC activator in the antibody inhibitory study but was one of the worst mAbs for extracting all five BoNT/B subtypes tested. This could be attributed to the relatively low affinity of mAb B1.1 for BoNT/B, or it may reflect either poor coupling of the mAb to the beads or inactivation of the mAb upon coupling.

Additionally, the extraction efficiency experiments demonstrated that some mAbs work very well for most subtypes, but not all of them. For example, mAb B11E8 yielded the highest or second highest response in terms of activity for BoNT/B1, /B2, /B3, and /B5, but was one of the worst choices for BoNT/B4. This mAb was not inhibitory for the activity of BoNT/B4; rather, this mAb has no affinity for BoNT/B4 compared to the other subtypes.

The Endopep-MS assay relies upon mAb extraction of BoNT from a clinical or food sample as a sample preparation step before analysis for the toxin, so high affinity, non-inhibitory mAbs are critical components of the assay. Additionally, mAbs that activate the enzymatic activity of the toxin after binding may further improve the sensitivity of detection in the Endopep-MS assay. Therefore, it is important to assess the binding affinities and potential enzymatic inhibition abilities of mAbs against a variety of BoNT subtypes within the chosen serotype before choosing mAbs for extraction. This assessment of a large panel of monoclonal BoNT/B antibodies should enable us to identify an antibody or a few mAbs that demonstrate strong extraction efficiency for all known BoNT/B, which currently includes the BoNT/B1-/B6 subtypes, without inhibiting the enzymatic activity of the toxin.

After testing a panel of 24 fully human antibodies against all BoNT/B subtypes in our possession, BoNT/B1-/B5, and examining both their inhibitory ability as well as their extraction efficiency, mAbs that had good results with all five subtypes were mAbs 1B18.1, 2B18.1, 2B18.2, 2B18.3, B12.2, and 2B23. Four of these mAbs (1B18.1, 2B18.1, 2B18.2, and 2B18.3) are clonally related, having almost the same HC variable regions and different LC variable regions, and binding to the same H_N _epitope. Antibodies interacting with all three domains of the toxin were represented, as B12.2 bound to the H_C _and 2B23 bound to the LC. It is known that using multiple mAbs which bind non-overlapping epitopes increases the effective affinity for the toxin by as much as 200-fold over the affinity of the individual antibodies [15]. Use of multiple mAbs binding different epitopes not only increases overall binding affinity, which is important for toxin extraction, but also offers a unique opportunity to design a mixture of mAbs that effectively bind a variety of epitopes, including regions that are conserved across the BoNT/B subtypes and also regions that may represent high-affinity binding sites for only a portion of the toxin subtypes. These multiple antibodies help to ensure that each toxin subtype will be recognized and extracted. As new BoNT/B subtypes are discovered, amino acid mutations affecting binding epitopes may be present. The use of multiple mAbs that recognize a variety of epitopes minimize the impact of these mutations, increasing confidence that all BoNT/B subtypes will be efficiently extracted.

1B18.1, 2B18.1, 2B18.2, and 2B18.3 bound the same epitope in the translocation domain. Examining their results in the extraction assay presented here, we found that 2B18.2 yielded the best overall results for subtypes BoNT/B1-/B5. Because of this, we are opting to use 2B18.2 as one of the mAbs for extraction of BoNT/B1-/B5 from sample matrices before analysis with the Endopep-MS method. Among the final list of mAbs which yielded excellent extraction results with all five subtypes tested, there were two binding unique epitopes which could be used: B12.2 and 2B23. B12.2 had better performance than 2B23, so we are opting to use B12.2, directed against the receptor binding portion of the HC, as a second mAb for extraction of BoNT/B1-/B5 from sample matrices before analysis with the Endopep-MS method. Unfortunately, BoNT/B6, the only other currently known BoNT/B subtype, was not available to us for testing. However, the sequence of BoNT/B6 for the epitope bound by 2B18.2 is completely conserved, so it would be anticipated that 2B18.2 could efficiently extract BoNT/B6 [19].

## Conclusions

In addition to determining the best mAbs for sample preparation before Endopep-MS, this work determined *in vitro *inhibition abilities of a panel of mAbs against BoNT/B1-/B5. Many mAbs showed similar results with BoNT/B1-/B5, but in some cases they differed, indicating differing toxin extraction efficiencies due to differing binding affinities or inhibition of toxin activity. In some cases, activation of toxin LC activity was seen. These findings indicate that mAb choice is crucial to the ability of these types of assays to sensitively detect a diverse range of BoNT/B toxin subtypes, which is a critical first step to providing proper treatment in a timely manner. In addition, these mAb characterizations have the potential to assist with mechanistic studies of BoNT/B protection and treatment, which is important for studying alternative therapeutics for botulism.

## Authors' contributions

SRK conceived of the study and participated in its design and coordination, and helped to draft the manuscript; WIS participated in testing the mAbs with Endopep-MS for extraction and inhibition; JL participated in producing and characterizing the mAbs used in this study and helped to draft the manuscript; ING participated in producing and characterizing the mAbs used in this study; CGR participated in producing and characterizing the mAbs used in this study and in epitope mapping; TJS conceived of the study, participated in its design and coordination, and helped to draft the manuscript; JDM conceived of the study, participated in its design and coordination, and helped to draft the manuscript; LAS participated in the study design and its coordination; JLP participated in the study design and its coordination; JRB conceived of the study and participated in its design and coordination, and helped to draft the manuscript. All authors read and approved the final manuscript.
